# Addressing insufficient physical activity in children with obesity in a 4-month physical activity on prescription intervention: a single-group pre–post study

**DOI:** 10.1186/s12889-026-29071-9

**Published:** 2026-08-20

**Authors:** Jessica Törnqvist, Katarina Lauruschkus, Susanne Bernhardsson, Karin Melin, Li Karlsson, Daniel Arvidsson, Jonatan Fridolfsson, Christian Greven, Stefan Lundqvist

**Affiliations:** 1 Centre for Physical Activity, Region Västra Götaland, Gothenburg, Sweden; 2 Unit of Physiotherapy, Department of Health and Rehabilitation, Institute of Neuroscience and Physiology, Sahlgrenska Academy, University of Gothenburg, Gothenburg, Sweden; 3 Faculty of Medicine, Department of Health Sciences, Lund University, Lund, Sweden; 4 Faculty of Health Sciences, Kristianstad University, Kristianstad, Sweden; 5 Research, Education, Development and Innovation, Primary Health Care, Region Västra Götaland, Gothenburg, Sweden; 6 Gillberg Neuropsychiatry Centre, Institute of Neuroscience and Physiology, Sahlgrenska Academy, University of Gothenburg, Gothenburg, Sweden; 7 Department of Paediatric Neurology and Psychiatry, Sahlgrenska University Hospital, Region Västra Götaland, Gothenburg, Sweden; 8 Centre for Health and Performance, Department of Food and Nutrition, and Sport Science, University of Gothenburg, Gothenburg, Sweden; 9 Center for Lifestyle Intervention, Department of Molecular and Acute Medicine, Sahlgrenska Academy, University of Gothenburg, Gothenburg, Sweden; 10 Department of MGAÖ, Sahlgrenska University Hospital, Region Västra Götaland, Gothenburg, Sweden

**Keywords:** Accelerometry, Paediatric obesity, Physical activity, Physical Activity on Prescription, Sedentary behaviour

## Abstract

**Background:**

Childhood obesity is a complex disease and a growing global public health challenge. Physical activity on prescription (PAP) is an evidence-based method to increase physical activity (PA) in adults but has been sparsely studied in children with obesity. The aim of the study was to explore changes in PA patterns in 6–12-year-old children with obesity after a 4-month PAP intervention and to explore age- and sex-related differences.

**Methods:**

The cohort consisted of 42 children (20 boys), 6–12 years old, treated at paediatric or rehabilitation clinics in Gothenburg and surrounding municipalities, Sweden. An individualised PAP intervention was provided, comprising individual counselling, a written prescription with an individually tailored PA recommendation, and structured follow-up. Main activities performed by the children were ball sports, swimming and walking, the latter including daily walks to and from school. Physical activity was measured using accelerometers attached to the participants’ hips for 7 days before and after the intervention. Changes pre- to post-intervention in PA patterns were analysed using Wilcoxon’s signed rank test.

**Results:**

Of the 42 included children, 35 completed the intervention (dropout rate 16.7%), of whom 32 provided valid accelerometer data. The sample was balanced by sex (16 girls/16 boys) and age (*n* = 16 in each subgroup). No significant changes were observed in PA patterns in the overall group or in subgroup analyses by sex and age. Overall satisfaction with the PAP treatment was high among both children and parents.

**Conclusions:**

Although no significant changes in PA patterns were observed post-intervention, children and parents expressed satisfaction with the PAP intervention. The lack of significant findings may partly be explained by the small sample size and limited statistical power, the extent and duration of the intervention, and the limited ability of accelerometers to capture certain types of activities. Further research is needed to explore the feasibility, adherence, and long-term effects of PAP interventions in children with obesity, with implications for broader public health efforts.

**Trial registration:**

ClinicalTrials.gov Identifier: NCT04847271, registered 14 April 2021.

**Supplementary Information:**

The online version contains supplementary material available at 10.1186/s12889-026-29071-9.

## Introduction

Childhood obesity is a major public health concern in most countries and tends to remain into adulthood with an increased risk of generating non-communicable diseases such as diabetes type 2, cardiovascular diseases, and premature death [1]. Globally, 8% of children and adolescents aged 5–19 was diagnosed with obesity in 2022 [2], and the prevalence of obesity is projected to increase to 18% in girls and 20% in boys by 2035 [3]. In Sweden, 7.2% of children aged 6–9 years were diagnosed with obesity and the prevalence of obesity in children (6–9 years) was 7.2% in 2022 [4]. Heredity as well as food habits, low levels of physical activity (PA) and increased sedentary behaviour (SED) are main factors associated with the development of obesity [1]. The complexity of obesity also involves biological, environmental and socioeconomical factors in addition to lifestyle [1].

The basis of all treatment for child obesity is structured interventions that combine lifestyle changes. Treatment should adopt a family-centred approach [1] to support healthier eating habits and increased PA [5]. Physical activity positively influences e.g. cardiorespiratory and muscular fitness, bone health, cardiometabolic health, and mental well-being [6–8]. It also plays a central role throughout life in maintaining weight balance [6]. Although increasing PA may not necessarily result in weight loss for children with obesity, and weight loss should not be the primary outcome, PA still provides important health benefits [5].

Physical activity is defined as any bodily movement produced by skeletal muscles that requires energy expenditure [9, 10]. Sedentary behaviour (SED) is characterised by energy expenditure of ≤ 1.5 metabolic equivalent of task (MET) while in a sitting, reclining or lying posture [11]. The World Health Organisation (WHO) and Swedish recommendations on PA and SED for children and adolescents include an average of 60 min of moderate-to-vigorous intensity PA (MVPA) per day [10, 12]. In addition to this, vigorous intensity PA (VPA) and muscle- and bone-strengthening activities should be performed at least three times a week. Children and adolescents with high levels of SED who do not meet PA recommendations should replace SED with more PA [10, 12].

Previous research shows that sex differences in PA emerge early, with boys being more active than girls, and that activity levels decline with age [13, 14]. Adolescent girls are the least likely to meet national PA recommendations, highlighting the importance of examining differences across sex and age groups. The recommendations for children and adolescents are based on relative intensity referring to self-perceived exertion [10]. Another way of measuring energy expenditure is using MET, which refers to an absolute intensity level of a specific PA where 1 MET represents an oxygen consumption at rest of 3.5 mL/min/kg [15]. Importantly, absolute and relative intensity may differ, as individuals with different fitness levels experience the same absolute intensity differently [16]. Therefore, objective measurements of PA using accelerometers are widely used to quantify activity intensity and volume, and have demonstrated good validity in paediatric and clinical populations, including children with obesity [15, 17].

Physical activity on prescription (PAP) is an evidence-based treatment method in Swedish health care, used for approximately 20 years, to increase PA in adults to prevent or treat various diseases [18, 19]. Swedish PAP is based on Social Cognitive Theory and the Transtheoretical Model [20] and includes three core components: individual counselling, a written prescription with tailored PA recommendation, and a structured follow-up [19, 21]. All licensed healthcare practitioners may offer PAP treatment, with recommendations adapted to the patients’ health status, preferences, and readiness to change [22]. National guidelines recommend PAP for patients not reaching 150 min of moderate intensity PA (MPA) per week [23]. A systematic review confirms the effectiveness of PAP in increasing PA among adults with insufficient activity levels [19].

Although PAP has been available in Swedish healthcare for more than two decades, its implementation in paediatric care remains underdeveloped and highly variable [24]. Previous research has shown that PAP can reduce BMI in children with obesity [25] and increase PA participation among children with cerebral palsy [26]. While the method is seen as acceptable, appropriate and feasible by healthcare professionals [26–28] its feasibility and effectiveness in increasing PA among children with obesity has not yet been investigated. Therefore, the aim of the study was to explore changes in PA patterns in 6–12-year-old children with obesity after a 4-month PAP intervention and to explore age- and sex-related differences.

## Methods

### Study design and setting

The study was part of the research project *“Implementation of physical activity on prescription project for children with obesity in paediatric healthcare (IMPA)”*, examining the feasibility of implementing PAP for children with obesity in paediatric health care [29]. The study was conducted in five paediatric clinics and three rehabilitation clinics in Gothenburg and surrounding municipalities. Usual care for children with obesity comprises multidisciplinary team treatment, with a strong emphasis on family support [5]. Combined lifestyle interventions form the cornerstone to support a healthy weight development. Families should receive support to facilitate behavioural changes in dietary habits, PA and SED. Care should be individually tailored, with annual follow-ups. Despite a scarcity of research on effects of PAP in children with obesity, national paediatric guidelines recommend the method as part of obesity treatment to motivate and increase PA [5].

Physical activity patterns were measured in children and parents at baseline, post-intervention, and 8 and 12 months after start of the intervention. The current study employed a single-group pre–post- design and reports post-intervention findings of participating children’s PA patterns. As this was a feasibility study, no formal a priori power calculation was performed. The target sample size of *n* = 30 was based on recommendations for feasibility research [30] and was considered sufficient to estimate key feasibility parameters to inform the design of a future definitive trial. The inclusion of a control group was not feasible due to practical, operational, and resource constraints of the study context. Reporting adhered to the TREND statement [31].

### Participants

Participants were consecutively recruited by healthcare professionals at the five included paediatric clinics and one of the rehabilitation clinics. Recruitment started in January 2022 and ended in May 2024. Although no formal sample size calculation was performed, the goal was to recruit about 60 children to allow for a potentially high attrition rate and to retain about 30 completers post-intervention. Recruitment was stopped before reaching the target of 60 children, for time and resource reasons and as experience from the earlier recruited participants showed that attrition was lower than anticipated. Inclusion criteria were children aged 6–12 years, diagnosed with obesity (ISO-BMI ≥ 30), PA levels below national recommendations [12], and a parent willing to participate. The PA criterion was determined by the recruiting healthcare professional based on parent-report of their child’s PA during the previous week. Exclusion criteria were severe psychiatric comorbidity (e.g., schizophrenia, psychosis, bipolar disorder), severe intellectual or physical disability, or planning to relocate outside the study area within 12 months.

### Intervention

The PAP intervention lasted four months and was adapted from the adult version to children with obesity based on barriers and facilitators identified by healthcare professionals in two previous studies [28, 32], as well as input from parents of children with obesity. Key adaptations included the use of visual aids, a stronger emphasis on family-centred support, and adaptation to the child’s social and cultural context. The intervention consisted of three core components delivered to all participants: an initial person-centred counselling session, a written PA prescription with individually tailored recommendations, and structured, individualised follow-up. During the initial counselling session, the healthcare professional, together with the child and parent, discussed current PA levels, preferences, barriers and facilitators, and jointly set goals for PA. The written prescription specified the frequency, duration, and intensity of the agreed activities. Based on this initial session, the intervention was further tailored and individualised to each child. Recommended activities could include both group-based and individual activities and were carried out in various settings such as school, home, outdoor environments, or through local sports associations. The choice of activities was guided by the child’s interests and context; examples included walking, cycling, swimming, and team sports. Seasonal variation was allowed for, enabling a mix of indoor and outdoor activities throughout the study period.

Follow-up was conducted regularly throughout the intervention and was individually adapted in terms of frequency, format, and content according to the child’s and family’s needs and preferences. Follow-up contacts aimed to support adherence, monitor progress, address barriers, and adjust the prescription when needed. These contacts were carried out by either a paediatric nurse, paediatrician, dietician, or physiotherapist, via face-to-face consultations at the paediatric or rehabilitation healthcare clinic, video consultation, telephone, and/or chat contact via a digital platform/mobile application. The mean number of registered consultations during the intervention, including initial PAP counselling, was 5.0 (SD 2.2, range 2–12). Participants had access to a project-developed mobile application that allowed them to view their PA prescription, register activities in an activity diary, and communicate with their healthcare provider. The application was used to support self-monitoring and adherence but was not synchronised with accelerometer data. Accelerometers were instead used only at predefined measurement points to provide objective assessments of PA, as continuous wear was not considered feasible in this population due to expected compliance challenges. As part of the intervention description, the most frequently reported activities in participants’ diaries were walking, ball sports, and swimming (Table 1).

**Table 1:** Activities registered in children’s activity diaries during the intervention

Everyday activities	*n*	Physical exercise	*n*
Walk to school	10	Ball sports	10
Walk from school	10	Swimming	10
Walking	9	Gymnastics	6
School physical education	8	Dance	2
Biking	7	Judo	2
Family activity	7	Horse riding	1
Play	6	Skating	1
Dog walk	3	Other	17
Other	5		

The reported activity count (n) is greater than the participant count, as the children often performed more than one activity

Before the intervention, participating healthcare professionals received brief standardised training covering the health effects of PA, the Swedish PAP concept, the adapted PAP intervention used in this study, study procedures, and the use of the digital support platform. As part of routine care for this patient group, dietary counselling was also provided.

### Outcomes

The primary outcome was time spent in MVPA. Secondary outcomes were time spent in low intensity PA (LPA) and SED. Data were collected at baseline and post-intervention (4 months after intervention start), using accelerometers (Axivity AX3, Axivity Ltd, UK). Participants were instructed to wear the accelerometer for seven consecutive days. The device was worn in an elastic belt around the waist over the right hip and was removed only when taking a shower or bath and, if necessary, during sleep. A valid measurement was defined as at least 10 registered hours per day for a minimum of two days. Although four valid days are commonly recommended [33], sensitivity analyses revealed no significant differences when applying this criterion; therefore, a minimum of two days was used to increase the number of participants with valid data. To complement accelerometer data, participants recorded bedtime, wakeup-time, and activities that are difficult to capture with accelerometery (e.g., resistance training, biking, and swimming) in an activity diary, either via a mobile application or on paper. These data were intended as supplementary information and were to be analysed separately from the objective accelerometer measures.

Accelerometers were initialised with a sampling frequency of 50 Hz and acceleration range expressed in gravity (g) of ± 8 g [34]. Raw data were processed using the 10 Hz frequency extended method [35], which has been shown to capture MVPA more accurately than traditional methods [36] and epoch length was set to three seconds to better capture the precise variation in PA [15] Intensity thresholds were defined using METs: SED as 1–1.5 METs, LPA as 1.6–3.9 METs [37], and MVPA as ≥ 4 METs, with MPA and VPA combined into a single MVPA category [16, 38].

Children’s and parents’ satisfaction with their PAP treatment was assessed using the Client Satisfaction Questionnaire (CSQ-8) which is a validated self-report instrument designed to measure patient satisfaction [35]. The questionnaire consists of 8 items, each with four response options with slightly different wording, ranging from 1= “poor/no, definitely not/quite dissatisfied” to 4= “excellent/yes, definitely/very satisfied”. Total score ranges from 8 to 32 points, with higher scores indicating greater satisfaction [35].

### Statistical analysis

Descriptive statistics are presented as absolute and relative numbers for categorical variables, and as means and standard deviations (SD) and median and interquartile range (IQR) for continuous variables. Unit of analysis was the individual participant. Due to skewed data, Wilcoxon signed rank test was used to analyse median changes from baseline to post-intervention of time in SED and in different PA levels. Baseline differences between sex and age groups were analysed using independent t-test and Mann-Whitney U test. Prespecified subgroup analyses were performed using Mann-Whitney U tests of differences in change between age (6 − 9 years vs. 10–12 years) and girls vs. boys. To assess robustness of the results, sensitivity analyses were performed using paired t-tests. Dropout analyses were conducted to compare baseline characteristics between completers and dropouts, using independent t-tests, Mann-Whitney U tests, and Fisher’s exact tests. Statistical significance was set to *p* < 0.05. Total scores on the CSQ-8 were obtained by summing all eight items with items 1, 3, 6 and 7 reverse-scored, and item and total scores were summarised descriptively. Five missing responses were imputed using the median of the remaining item responses for the respective individual. No imputation was performed for accelerometer-derived outcomes; missing data were handled using complete case analysis. Statistical analyses were performed using IBM SPSS Statistics (version 28).

### Ethical considerations

Participants received oral and written information by the project coordinator at each clinic and/or by a research team member. The written information was available in child and parent versions in five languages. Other study documents, including accelerometer instructions and activity diary, were also available in different language versions and a budget allowance for interpreter was provided, to enable participation of non-Swedish speaking children.

## Results

### Participant characteristics

Forty-two children (20 boys) were included in the study, with a mean age of 9.6 (SD 2.0) years and mean BMI-SDS of 3.0 (SD 0.4, range 2.0 − 3.9) (a value of > 2.0 indicates obesity). As there were two pairs of siblings included, the number of participating parents was 40. The mean age of the participating parent was 41.8 (SD 6.1) years. Just over half (55%) were mothers and 51% had completed secondary education. Approximately one third (32%) held a university degree, while about 17% had completed only primary education or less. Nearly half of the parents (48%) were born outside Sweden. For six of the children who completed the intervention (17.1%) and their parents, Swedish language proficiency was limited, and an interpreter was required during study visits. There were no significant differences between participants who completed the intervention and dropouts. Baseline characteristics for the participating children are presented in Table 2 and those for the parents in Table 3.

**Table 2:** Baseline characteristics of participating children

Variables	Total (*n* = 42)	Boys (*n* = 20)	Girls (*n* = 22)	*P* value	6–9-year-old (*n* = 21)	10–12-year-old (*n* = 21)	*P* value
Age, years^a^	9.6 (2.0)	10.2 (2.1)	9.2 (1.9)		8.0 (1.2)	11.4 (1.0)	
BMI-SDS^a^	3.0 (0.4)	3.1 (0.4)	2.9 (0.4)		3.0 (0.5)	2.9 (0.4)	
Comorbid diagnoses^c^ (%)	18 (43)						
SED, min/day^a^	588 (90)	624 (87)	556 (81)	0.012^d^	554 (102)	662 (61)	0.012^d^
SED, min/day^b^	589 (525–658)	651 (572–682)	566 (485–614)	0.014^e^	558 (454–634)	626 (578–664)	0.026^e^
LPA, min/day^a^	233 (59)	212 (55)	252 (58)	0.026^d^	261 (59)	206 (46)	0.002^d^
LPA, min/day^b^	222 (187–280)	199 (178–247)	248 (193–308)	0.037^e^	250 (220–308)	197 (178–235)	0.002^e^
MVPA, min/day^a^	44 (20)	40 (22)	47 (17)	0.249^d^	45 (18)	42 (22)	0.664^d^
MVPA, min/day^b^	42 (33–53)	38 (24–50)	44 (36–55)	0.158^e^	40 (33–55)	44 (26–49)	0.399^e^

*BMI-SDS *body mass index-standard deviation score, *SED *sedentary behaviour, *LPA *low-intensity physical activity, *MVPA *moderate-to-vigorous-intensity physical activity

^a^Values are given as mean (standard deviation)

^b^Values are given as median (interquartile range)

^c^Most common comorbidities; neuropsychiatry (14%), asthma (12%), and allergy (10%)

*P*-values were determined by an ^d^Independent samples t-test or a ^e^Mann-Whitney U-test

Statistical significance was set at *p* < 0.05

**Table 3:** Characteristics of parents

	Participating parent (*n* = 40)	Other parent (*n* = 39)
Age, years^a^	41.8 (6.1)	42.1 (7.7)
Sex^b^		
Male	18 (45)	22 (56.4)
Female	22 (55)	17 (43.6)
Country of birth^b^		
Sweden	21 (52.5)	18 (46.2)
Other European countries	7 (17.5)	9 (23.1)
Outside Europe	12 (30.0)	12 (30.7)
Education^b^		
Primary school or less (≤ 9 years)	6 (17.1)	5 (12.8)
Secondary school	21 (51.2)	23 (59.0)
University	13 (31.7)	11 (28.2)
Occupation^b^	(*n* = 39)	(*n* = 37)
Blue collar profession	22 (56.4)	23 (62.2)
White collar profession	14 (35.9)	9 (24.3)
Other^c^	3 (7.7)	5 (13.5)

^a^Values are given as mean (standard deviation)

^b^Values are given as number (percent)

^c^Other includes students, unemployed, or on sick leave

### Post-intervention assessment

**Fig. 1:**
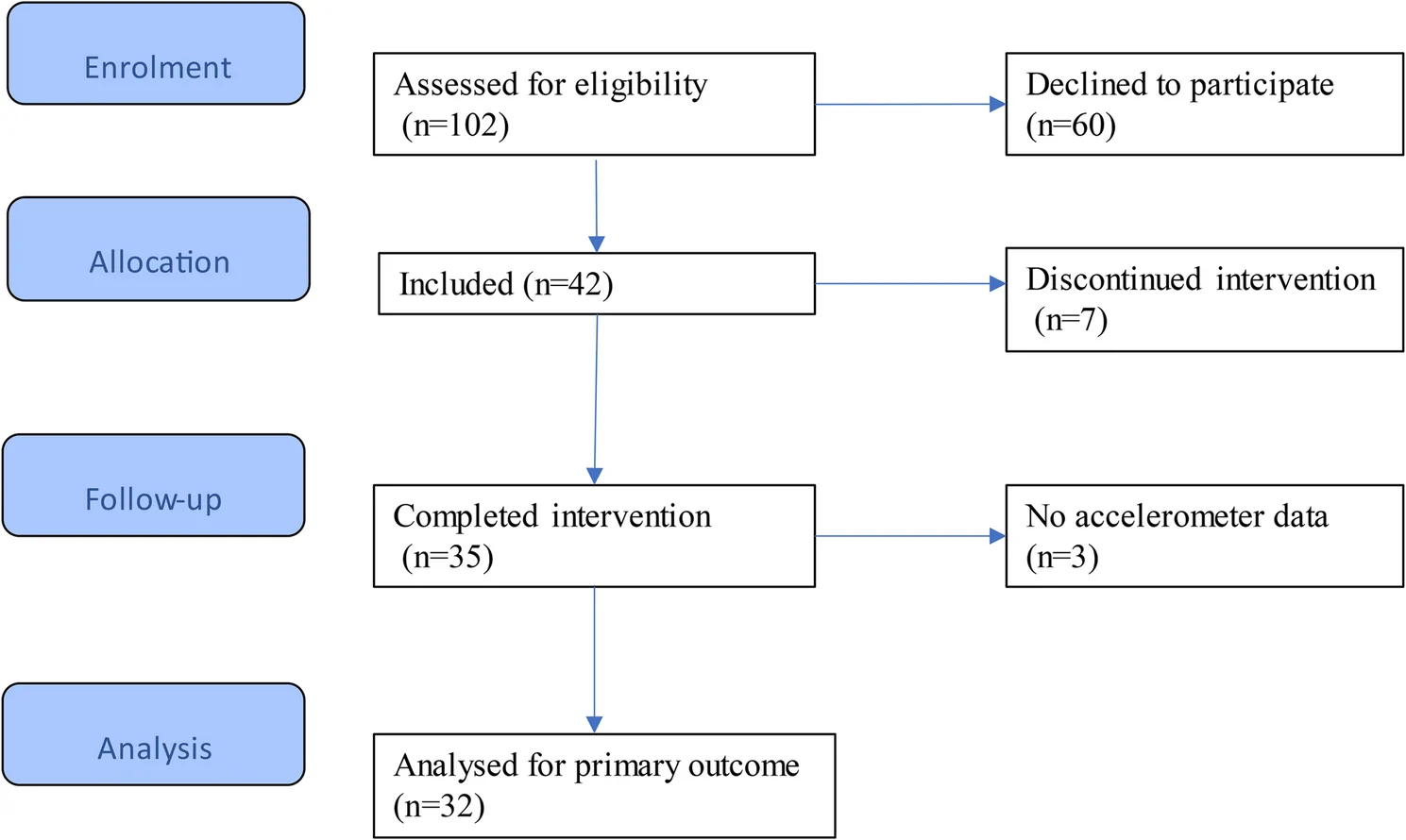
Patients flow through study according to CONSORT

All 42 children enrolled in the study provided valid accelerometer data at baseline. Post-intervention, 35 children remained (dropout rate 16.7%). Three children declined to wear the accelerometer, leaving 32 who underwent accelerometer measurement and were included in the analyses (Fig. 1). All accelerometer measurements performed were considered valid.

At baseline, the median time spent in SED was 589 min per day (IQR 543–664) compared with 634 min per day (IQR 545–701) post-intervention. Median time spent in LPA was 222 min per day (IQR 187–274) at baseline and 215 min per day (IQR 183–256) post-intervention. For MVPA, the median was 44 min per day (IQR 32–54) at baseline compared to 36 min per day (IQR 28–44) post-intervention. None of the changes were statistically significant (Table 4).

**Table 4:** Outcomes of sedentary behaviour, low and moderate-to-vigorous intensity physical activity (minutes/day) at baseline and post-intervention

Variable	Baseline median (IQR) (*n* = 32)	Post-intervention median (IQR) (*n* = 32)	*p*-value
SED			
Total group	589 (543–664)	634 (545–701)	0.210^a^
Sex			0.546^b^
Boys (*n* = 16)	655 (572–696)	667 (654–728)	0.215^a^
Girls (*n* = 16)	574 (482–608)	584 (498–616)	0.796^a^
Age			0.291^b^
6–9 years (*n* = 16)	571 (461–678)	584 (485–662)	0.959^a^
10–12 years (*n* = 16)	619 (575–664)	662 (602–731)	0.121^a^
LPA			
Total group	222 (187–274)	215 (183–256)	0.191^a^
Sex			0.851^b^
Boys (*n* = 16)	197 (178–243)	184 (167–244)	0.408ª
Girls (*n* = 16)	248 (198–308)	223 (209–275)	0.255ª
Age			0.940^b^
6–9 years (*n* = 16)	250 (202–302)	233 (194–281)	0.215ª
10–12 years (*n* = 16)	197 (180–240)	205 (173–226)	0.469ª
MVPA			
Total group	44 (32–54)	36 (28–44)	0.130^a^
Sex			0.451^b^
Boys (*n* = 16)	35 (23–50)	35 (27–42)	0.796ª
Girls (*n* = 16)	46 (38–59)	40 (30–44)	0.098ª
Age			0.083^b^
6–9 years (*n* = 16)	40 (32–55)	41 (30–46)	0.836ª
10–12 years (*n* = 16)	45 (28–51)	35 (27–40)	0.056ª

*IQR *interquartile range, *SED *sedentary behaviour (1–1.5 METs), *LPA *low intensity physical activity (1.6–3.9 METs), *MVPA *moderate-to-vigorous intensity physical activity (≥ 4 METs)

*P*-values were determined by ^a^Wilcoxon signed rank test in within-group analyses or ^b^Mann-Whitney U test in between-group analyses

Statistical significance was set at *p* < 0.05

The subgroup analyses showed no significant differences between boys and girls or between younger (6–9 years) and older (10–12 years) children (Table 4). Sensitivity analyses yielded similar, non-significant, results as the main analyses. In addition to accelerometer data, most children reported biking and/or swimming (not analysed due to missing, incomplete or incorrect data).

Thirty-four children and 35 parents provided complete CSQ-8 data. All item responses were negatively skewed. Two values in the children’s scores and three of parents’ scores were missing and were imputed. Overall satisfaction with the PAP treatment received was high among both children and parents, with 91.2% and 82.8%, respectively, being mostly or very satisfied. Median CSQ-8 score was 28.5 (IQR 24.0–31.0) of a total of 32 among children and 28.0 (IQR 24.0–30.0) among parents. No responses were recorded in the lowest response category for most items, indicating an absence of strong dissatisfaction. The highest ratings were observed for willingness to recommend the treatment. All item-level responses are presented in Additional file 1. No adverse events during or after the intervention were reported by any of the participants.

## Discussion

The main findings of this study indicate that there were no significant changes in accelerometer-measured PA patterns following the 4-month PAP intervention in 6–12-year-old children with obesity, nor in the subgroup analyses by sex and age. However, both children and their parents were generally satisfied with the PAP treatment.

Before the intervention, children in our study spent a median of 44 min per day in MVPA, which is consistent with a previous study on Spanish children with abdominal obesity that also reported 44 min of MVPA per day [39]. In contrast, international registry data reported a higher mean MVPA of 52 min per day [40]—likely due to the dataset also including normal-weight children who generally engage in more MVPA [41]. In the only previous study we are aware of that has evaluated PA in relation to a Swedish PAP intervention in a paediatric population, conducted in children with cerebral palsy, median MVPA was considerably higher at 84 min per day but varied greatly; children with severe motor limitations averaged only 28 min per day [26].

Post-intervention, the children in our study spent a median of 36 min per day in MVPA, representing a decrease of eight minutes compared to baseline. One possible explanation for the lack of significant change is that participants may have been motivated to increase their PA levels already upon study enrolment, which could have led to inflated baseline estimates. Given the limited number of studies evaluating PAP in children, comparisons were primarily made to studies of other lifestyle interventions employing accelerometry in order to provide context. Similar patterns have been reported in previous research after various types of lifestyle interventions, often comprising multiple components. A systematic review of children with overweight and obesity aged 6–11 years reported only a negligible increase in MVPA of 0.76 min per day [42]. Similarly, interventions delivered during out-of-school hours for children aged 4–12 years, both with and without obesity, demonstrated minimal influence on total daily MVPA, a mean of 1.7 min per day more than in controls [43]. Furthermore, a systematic review of school-based programmes, also in mixed populations, reported a small but statistically significant effect on MVPA in favour of the control group, and no impact on SED [44].

However, other studies have shown more promising results. The previously mentioned PAP study among children with cerebral palsy reported a median increase in MVPA from 84 to 106 min per day at the 8-month follow-up [26], while a Spanish study observed a significant improvement in MVPA from 44 to 49 min per day after an 8-week multidisciplinary lifestyle intervention, although time spent in LPA decreased in both intervention and control groups [39]. Furthermore, a systematic review and meta-analysis concluded that lifestyle interventions incorporating PA for children 6–12 years old with obesity can lead to significant increases in MVPA [45].

Collectively, findings from previous studies highlight the considerable challenges in promoting changes in PA among children, particularly those with obesity, as childhood obesity is a multifaceted condition shaped by biological, behavioural, and environmental influences. This complexity may partly explain the lack of improvement and the observed decrease in MVPA in the present study, as even interventions with multiple components have shown limited effects on daily PA. In addition, the relatively short duration of the intervention and the individualised but variable extent of support may have limited its impact on sustained behaviour change. Research evaluating PAP for children remains scarce, and existing lifestyle interventions vary widely in design, duration and components, resulting in highly heterogenous outcomes. This variability, together with the modest effects reported in previous studies, makes it difficult to draw firm conclusions regarding the effectiveness of such approaches in promoting PA among children with obesity. It also underscores the need for future research to examine how personalised strategies, such as individualised support, can be optimised to enhance engagement and address the complex determinants of PA in this population.

We observed no significant differences in PA levels between sex or age groups, whereas previous research has shown that boys are generally more physically active than girls of the same age [13], and that time spent in MVPA typically peaks around seven years of age before gradually declining between ages seven and fourteen [14]. Boys are also reported to have higher mean levels of MVPA, while differences between boys and girls in SED and LPA appear minimal or absent [46], which contrasts with the findings of our study. The absence of subgroup differences in the present study may be partly due to the relatively small sample size. However, it is also possible that baseline differences in LPA and SED between subgroups influenced the results, such that similar changes over time may have obscured meaningful group differences. This possibility should be considered when interpreting the null subgroup findings.

The non-significant changes in PA patterns among children in this study may have been influenced by several factors. The four-month intervention period may have been too short to produce measurable changes in PA and SED. The varying and sometimes low number of follow-up consultations may have been insufficient to adequately support and promote behaviour change. Participant recruitment proved challenging and had to be extended by a year, largely due to the COVID-19 pandemic and associated resource and staffing constraints. At the same time, healthcare professionals had limited opportunity for training in the PAP method, and the participating clinics reevaluated and revised their obesity management and clinical pathways, resulting in more children being referred to other services. These circumstances likely affected both the recruitment process and the PAP intervention, including the capacity to conduct follow-up.

Accelerometers are commonly used for objective measurement of PA and are generally considered to have high validity [15]. However, methodological decisions in data collection and processing may substantially influence the outcome [15]. In this study, the choice of a 4 MET threshold to define MVPA, rather than the more traditional 3 MET cut-off [47], likely affected the amount of time classified as MVPA and may have reduced sensitivity to detect changes in this population with generally low PA levels. A 3 MET threshold corresponds to relatively low-intensity activities, such as walking at approximately 3–4 km/h and may therefore be more appropriate for capturing meaningful changes in populations with low baseline activity levels. Furthermore, in children with obesity, lower absolute MET levels may still represent higher relative intensity, which further complicates the interpretation of intensity thresholds. The selected higher cut-off was based on previous findings indicating that PA at the upper range of MPA is associated with favourable health outcomes, including lower BMI in children [34] and improved cardiometabolic health in adults [16]. Nevertheless, the use of this higher threshold may have led to an underestimation of meaningful changes in PA.

In addition, the criteria for valid accelerometer data may have influenced the results. While sensitivity analyses showed no differences between two and four valid days, requiring only two days may still reduce measurement reliability and increase variability relative to commonly recommended protocols, potentially limiting the ability to detect true pre–post changes in PA. More broadly, accelerometer-based assessments capture only a limited snapshot of habitual PA, and even protocols with more valid days may not fully reflect day-to-day variability in children’s behaviour. These methodological constraints should be considered when interpreting the findings. The known limitations of hip-worn accelerometers to capture activities such as resistance training, swimming and biking, which were self-reported by the participants to some extent, suggest that the total level of PA may have been underestimated. Nevertheless, hip placement is generally considered one of the most suitable options for capturing movement patterns in children [15].

The Swedish National Board of Health and Welfare emphasises that children with obesity have the right to a diagnosis and access to the best available treatment [5], which requires adequate knowledge and resources to tailor interventions to each child. Although the participating healthcare professionals in this study received brief training, limited familiarity with PAP and its core components may have led to less precise matching of PA recommendations to the child’s abilities, preferences, and readiness to change, potentially reducing adherence and attenuating intervention effects. Organisational constraints, such as limited time, may have further restricted opportunities for adequate support, follow-up, and behavioural guidance. Experiences from PAP implementation in school settings have shown that the intervention is often perceived as a complex and challenging process, where successful promotion of PA relies on addressing the child’s broader social environment [27]. Although the present study was conducted in specialised paediatric and rehabilitation clinics, those findings may still be relevant, as many of the same contextual factors influence outcomes across settings. The need to consider family involvement, accessibility and costs of activities, as well as helping children identify enjoyable and meaningful forms of movement, are central in both school and clinical contexts [27]. However, in specialised care, children may present with more complex needs and lower baseline PA, which may further increase the importance of tailored support and interdisciplinary approaches.

Despite its limitations, a main strength of this study was the use of accelerometers to assess PA. This method is considered the gold standard of measuring PA, with high objectivity and validity [17] compared to self-reported PA [15]. Another strength was that the PAP intervention was carried out in everyday clinical practice, without any additional resources and mimicking standard care, potentially increasing generalisability of the findings. Including families with a non-Swedish origin implies that our sample represents a cultural, ethnic, and socioeconomic diversity that mirrors current population demographics in Sweden today, further enhancing generalisability.

Main limitations of this study include the small sample size, lack of a control group, and the accelerometer’s inability to capture all types of PA. The absence of a control group limits the ability to attribute observed changes, or lack thereof, specifically to the intervention, as natural variations over time or external factors may also have influenced the results. This reduces the internal validity and makes it more difficult to draw firm conclusions regarding intervention effects. The small sample size further limits the statistical power of the study and increases the risk of a type II error, meaning that potentially meaningful changes in PA may not have been detected. This is particularly relevant given the non-significant findings. In addition, as this was a feasibility study, no formal sample size calculation was performed, and the final sample may therefore have been underpowered, especially for detecting small effects or conducting subgroup analyses. The dropout rate of 16.7% may be considered relatively high. However, the study was conducted during and immediately after the COVID-19 pandemic, a period characterised by increased sickness absence among children, parents, and healthcare professionals, which may partly explain the attrition. Another possible dropout reason was that some children felt uncomfortable or embarrassed about wearing the accelerometer belt for an entire week, indicating that alternative devices, placements, or measurement approaches may be needed in future research.

Future research should examine the PAP treatment process and intervention fidelity to its core components for children with obesity. Although the present intervention included adaptations from the adult PAP model, such as family involvement, person-centred counselling, and individualised prescriptions based on each child’s preferences, context, and barriers, further refinement and evaluation of these components are needed. To achieve and sustain behavioural change in PA, PAP may need to be more systematically tailored to the child’s developmental stage and social context and evaluated over the long term. It is essential that the individualised PA plan is optimally designed regarding dose, enjoyment, and regular follow-up by healthcare professionals over an extended period. Furthermore, support from adults in the child’s environment, within the family, at school, and during leisure activities, needs to be strengthened to promote engagement in regular PA. In addition, health-economic analyses are needed to evaluate the costs and benefits of PAP and behaviour change. It is also important to further explore children’s and parents’ experiences of receiving an adapted PAP intervention.

## Conclusions

This study contributes to new knowledge about using PAP as an intervention in paediatric healthcare for children with obesity. Although the study showed no significant changes in PA patterns after a four-month PAP intervention, both children and parents expressed satisfaction with the PAP treatment. The small sample size, the varied extent of PAP support and follow-up provided by the healthcare professionals, and the limited ability for the accelerometer to capture specific activities may have affected the results. Future research should explore feasibility, adherence and long-term effects of PAP interventions on PA patterns in children with obesity.

## Supplementary Information

Supplementary Material 1: Frequency distribution, mean and median scores of children’s and parents’ satisfaction with the PAP treatment.

## Data Availability

The dataset and materials used and/or analysed in the current study are available from the corresponding author upon reasonable request.

## Abbreviations

BMI: Body mass index
BMI-SDS: Body mass index standard deviation score
CSQ: Client satisfaction questionnaire
g: gravity
Hz: Hertz
IMPA: Implementation of physical activity on prescription
IQR: Interquartile range
LPA: Low intensity physical activity
MET: Metabolic equivalent of task
MPA: Moderate intensity physical activity
MVPA: Moderate-to-vigorous intensity physical activity
PA: Physical activity
PAP: Physical activity on prescription
SD: Standard deviation
SED: Sedentary behaviour
VPA: Vigorous intensity physical activity
WHO: World health organisation
