# Supplementary material for: Addressing insufficient physical activity in children with obesity in a 4-month physical activity on prescription intervention: a single-group pre–post study
Source: BMC Public Health. 2026 Aug 20;26:2407. doi: 10.1186/s12889-026-29071-9 (PMC13495397; doi:10.1186/s12889-026-29071-9)
Supplement: Supplementary file 1 [file 12889_2026_29071_MOESM1_ESM.doc]

**Additional file 1** Frequency distribution, mean and median scores of children’s and parents’ satisfaction with the PAP treatment

| **Item / Total** | **Response 1  n (%)** | **Response 2  n (%)** | **Response 3  n (%)** | **Response 4  n (%)** | **Mean (SD)** | **Median  (IQR)** |
| --- | --- | --- | --- | --- | --- | --- |
| **Children’s scores (n=34)** |  |  |  |  |  |  |
| 1. How would you rate the quality of treatment you received? | - | 4 (11.8) | 13 (38.2) | 17 (50.0) | 3.4 (0.7) | 3.5 (3.0–4.0) |
| 2. Did you get the kind of treatment you wanted? | 1 (2.9) | 4 (11.8) | 15 (44.1) | 14 (41.2) | 3.2 (0.8) | 3.0 (3.0–4.0) |
| 3. To what extent has the treatment met your needs? | — | 4 (11.8) | 16 (47.1) | 14 (41.2) | 3.3 (0.7) | 3.0 (3.0–4.0) |
| 4. If a friend were in need of similar help, would you recommend the treatment? | — | 1 (2.9) | 13 (38.2) | 20 (58.8) | 3.6 (0.6) | 4.0 (3.0–4.0) |
| 5. How satisfied are you with the amount of help you received? | — | 4 (11.8) | 9 (26.5) | 21 (61.8) | 3.5 (0.7) | 4.0 (3.0–4.0) |
| 6. Has the treatment you received helped you to deal more effectively with your problems? |  | 2 (5.9) | 16 (47.1) | 16 (47.1) | 3.4 (0.6) | 3.0 (3.0–4.0) |
| 7. Overall, how satisfied are you with the treatment you received? | — | 3 (8.8) | 15 (44.1) | 16 (47.1) | 3.4 (0.6) | 3.0 (3.0–4.0) |
| 8. If you were to seek help again, would you come back to our service? | — | 3 (8.8) | 13 (38.2) | 18 (52.9) | 3.4 (0.7) | 4.0 (3.0–4.0) |
| **Children’s total CSQ-8 score** |  |  |  |  | 27.2 (4.4) | 28.5 (24.0–31.0) |
| **Parents’ scores (n=35)** |  |  |  |  |  |  |
| 1. How would you rate the quality of the treatment your child received? | — | 7 (20.0) | 15 (42.9) | 13 (37.1) | 3.2 (0.7) | 3.0 (3.0–4.0) |
| 2. Did your child get the kind of treatment you wanted? | — | 6 (17.1) | 10 (28.6) | 19 (54.3) | 3.4 (0.8) | 4.0 (3.0–4.0) |
| 3. To what extent has the treatment met your child’s needs? | 1 (2.9) | 7 (20.0) | 16 (45.7) | 11 (31.4) | 3.1 (0.8) | 3.0 (3.0–4.0) |
| 4. If a friend’s child were in need of similar help, would you recommend the treatment? | — | 3 (8.6) | 7 (20.0) | 25 (71.4) | 3.6 (0.6) | 4.0 (3.0–4.0) |
| 5. How satisfied are you with the amount of help your child received? | — | 5 (14.3) | 11 (31.4) | 19 (54.3) | 3.4 (0.7) | 4.0 (3.0–4.0) |
| 6. Has the treatment your child received helped him/her to deal more effectively with his/her problems? |  | 4 (11.4) | 22 (62.9) | 9 (25.7) | 3.1 (0.6) | 3.0 (3.0–4.0) |
| 7. Overall, how satisfied are you with the treatment your child received? | — | 6 (17.1) | 11 (31.4) | 18 (51.4) | 3.3 (0.8) | 4.0 (3.0–4.0) |
| 8. If you were to seek help again for your child, would you come back to our service? | 1 (2.9) | 6 (17.1) | 8 (22.9) | 20 (57.1) | 3.3 (0.9) | 4.0 (3.0–4.0) |
| **Parents’ total CSQ-8 score** |  |  |  |  | 26.4 (5.1) | 28.0 (24.0–30.0) |

CSQ-8: Client Satisfaction Questionnaire, 8 items; IQR: Interquartile range
